# Molecular Regulation of Cancer Cell Migration, Invasion, and Metastasis

**DOI:** 10.1155/2019/1356508

**Published:** 2019-05-14

**Authors:** Lubna Tahtamouni, Mamoun Ahram, Jennifer Koblinski, Christian Rolfo

**Affiliations:** ^1^Hashemite University, Zarqa, Jordan; ^2^University of Jordan, Amman, Jordan; ^3^Virginia Commonwealth University, Richmond, USA; ^4^University of Maryland School of Medicine, Baltimore, MD, USA

Cancer metastasis represents an advanced stage of malignancy and is the leading cause of cancer-related deaths. Metastasis is a multistep process that includes migration and invasion of cancer cells, hallmarks of malignancy. These processes require the involvement of a wide array of cellular mechanisms led by cytoskeleton dynamics as well as molecular alterations such as expression of adhesion and proteolytic enzymes. Cell migration in itself is a highly integrated process that includes development of cytoplasmic protrusions, attachment, and traction. Additional secondary changes associated with cell migration and invasion include production of reactive oxygen species (ROS), development of chemoresistant cancer stem cells, introduction of mutations in DNA damage repair genes, and contribution of microRNAs (miRNAs). Cell release of exosomes and their participation in modulating cell behavior are a new and exciting observation. In this special issue on Molecular Regulation of Cancer Cell Migration, Invasion, and Metastasis, we have invited a few papers that address such issues.

One of the papers investigated the binding partner of AG73, a synthetic laminin *α*1 chain peptide, in breast cancer cells that mediate cancer progression. The findings demonstrated an intrinsic interaction between AG73 and syndecans that mediates tumor cell adhesion and invasion. One of the papers investigated the expression of syndecans, Sdc1 in particular, in tumor cells and stroma of invasive lobular and ductal breast carcinoma. Sdc1 was expressed in the epithelium of 90% carcinoma of both histological types. It was also most frequently expressed in tumor-associated stroma. In metastatic epithelium, Sdc1 was negatively correlated with patient's age and statuses of estrogen receptors (ERs) and progesterone receptors (PRs) in the primary tumors, while stroma of metastases demonstrated a positive correlation with focus number in primary tumors and a negative association with PRs in primary tumors. One of the papers found a role of syndecan-2 in colorectal cancer cell migration. This finding can help understand the biology of tumors and suggests that syndecan-2 may be used as a target to therapy and diagnosis.

One of the papers of this special issue assessed the expression level of Epac1 and PDE4 in rectal carcinoma. It was found that their expression is increased in rectal carcinoma tissue; however, no significant association between these proteins and degree of differentiation, histological type, and lymph node metastasis was reported. The fifth paper investigated the anticancer potential of metformin against glioblastoma multiforme cancer. The results showed a significant decrease in survival, motility, and invasion as well as an increase in cell adhesion in metformin-treated cancer cells.

One of the papers assessed the role of long noncoding RNA LOXL1-AS1 on human medulloblastoma cell proliferation and metastasis. The paper reported overexpression of Lcn RNA LOX1-AS1 in medulloblastoma tissues as well as in advanced stages. The proliferation and metastasis of medulloblastoma by this noncoding RNA was mediated by activation of the P13K-AKT pathway. The latter study opens the door for the exciting roles of noncoding RNA in regulating cancer cell behavior.

